# The effect of head orientation on vestibular signal-based modulation of paraspinal muscle activity during walking

**DOI:** 10.1007/s00421-024-05620-1

**Published:** 2024-10-04

**Authors:** Yiyuan C. Li, Koen K. Lemaire, Sjoerd M. Bruijn, Simon Brumagne, Jaap H. van Dieën

**Affiliations:** 1 Department of Human Movement Sciences, Faculty of Behavioral and Movement Sciences, Vrije Universiteit Amsterdam, Amsterdam Movement Sciences, van der Boechorststraat 9, 1081 Amsterdam, The Netherlands; 2 Department of Rehabilitation Sciences, KU Leuven, Louvain, Belgium

**Keywords:** Trunk control, Vestibulospinal reflexes, Paraspinal muscle, Electrical vestibular stimulation, Head orientation

## Abstract

**Background:**

Vestibulospinal reflexes play a role in maintaining the upright posture of the trunk. Head orientation has been shown to modify the vestibulospinal reflexes during standing. This study investigated how vestibular signals affect paraspinal muscle activity during walking, and whether head orientation changes these effects.

**Methods:**

Sixteen participants were instructed to walk on a treadmill for 8 min at 78 steps/min and 2.8 km/h in four conditions defined by the presence of electrical vestibular stimulation (EVS) and by head orientation (facing forward and facing leftward), while bipolar electromyography (EMG) was recorded bilaterally from the paraspinal muscles from cervical to lumbar levels.

**Results:**

In both head orientations, significant phasic EVS-EMG coherence in the paraspinal muscles was observed at ipsilateral and/or contralateral heel strikes. Compared to walking with the head forward, a significant decrease was found in EVS-evoked responses (i.e., EVS-EMG coherence and gain) when participants walked with the leftward head orientation, with which EVS induced disturbance in the sagittal plane. This overall decrease can be explained by less need of feedback control for walking stabilization in the sagittal plane compared to in the frontal plane. The decrease in coherence was only significant at the left lower vertebral levels and at the right upper vertebral levels around left heel strikes.

**Conclusion:**

These findings confirm the contribution of the vestibular afferent signals to the control of paraspinal muscle activity during walking and indicate that this control is changed in response to different head orientations.

**Graphical abstract:**

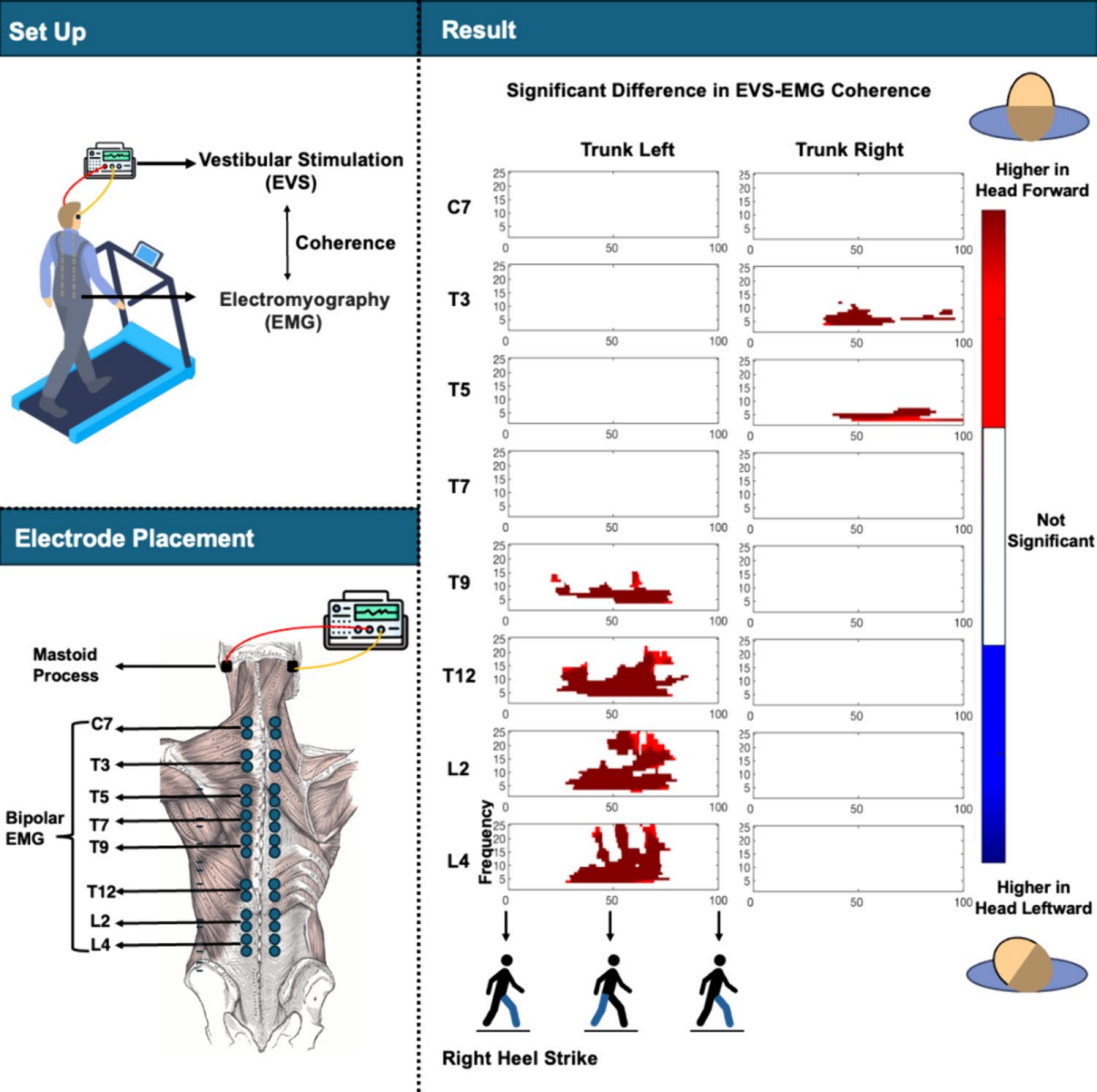

## Introduction

Controlling the orientation of the trunk is imperative to prevent falling when walking. With its large mass and relatively high location above the base of support, trunk has a large potential energy. Consequently, a small deviation from its upright orientation can produce a substantial destabilizing moment. Maintaining the upright posture relies on feedback control, based on integrated visual, vestibular, proprioceptive and tactile inputs (Peterka 2002; Goodworth and Peterka 2009; Maaswinkel et al. 2014; Andreopoulou et al. 2015; Cofre Lizama et al. 2016; van Drunen et al. 2016). Disturbances of the vestibular system through electrical vestibular stimulation (EVS) have been widely used to investigate the contribution of vestibular signals to postural control (Britton et al. 1993; Ali et al. 2003; Iles et al. 2007; Dakin et al. 2010; Forbes et al. 2013; Magnani et al. 2021). This stimulation, a small electrical current, activates the afferent nerve fibers of the vestibular organs and produces an illusion of self-movement, as a consequence of which a body sway opposite to the illusory movement is induced (Lund and Broberg 1983; Mian and Day 2009).

Coherence is a frequency-domain measure of linear association between the signals. It varies between 0, indicating no association, and 1, indicating perfect association. EVS-EMG coherence thus indicates how strong muscle activity is coupled to afferent signals from the vestibular system. If the coupling is strong (i.e., coherence is significant), the frequency response function between the EVS signal and electromyography (EMG) activity can be estimated, which provides information on the gain and delay of the feedback from the vestibular system to muscle activity. Previously, we studied the coherence between EVS and paraspinal muscle activity from the cervical to the lumbar vertebra level while walking on the treadmill with the head facing forward. We showed that vestibular signals modulated paraspinal muscle activity (Li et al. 2024). Peak coherence was found at ipsilateral heel strike at lower vertebral levels, whereas it occurred at the contralateral heel strike at higher vertebral levels. These couplings appeared to be functional in maintaining an upright trunk orientation in the frontal plane.

In standing, EVS-induced responses have been demonstrated to be craniocentric (Lund and Broberg 1983; Mian and Day 2014). For example, with the anode placed on the right mastoid, a rightward body sway will be induced by EVS when facing forward, while a backward sway will be evoked when the head is turned 90° to the right (Lund and Broberg 1983; Ali et al. 2003). A study by Forbes et al. reported that EVS-EMG coherence in the soleus muscle was maximal with the head facing 90° to the left, while it diminished progressively when participants rotated their head towards the right, and finally decreased to zero when facing forward (Forbes et al. 2016). Thus, the direction of EVS-induced disturbances can be manipulated by changing head orientation.

Head orientation also changes the effect of EVS on paraspinal muscle activity in standing. When facing sideways, EVS induced disturbances in the sagittal plane, with synergistic activity of the bilateral paraspinal muscles. Facing forward, EVS induced disturbances in the frontal plane, with bilateral paraspinal muscles acting as antagonists (Ali et al. 2003). To our knowledge, it is unknown if, and how, paraspinal muscles responses to EVS change as a consequence of head orientation during walking.

In this study, we investigated whether- and how vestibular signals influence paraspinal muscle activity during walking with the head facing forward and sideways. We expected that when facing sideways, illusion of self-movement would be induced mostly in the sagittal plane, causing the left and right paraspinal muscles to respond synergistically. Consequently, we hypothesized that EVS-EMG coherence would show a more symmetric pattern between the left and right sides of the trunk and among the upper to lower vertebral levels, compared to facing forward. No impact of head orientation on the delay of the feedback from the vestibular system to muscle activity was expected.

## Methods

Part of the data presented in this study were reported earlier by Li et al. (Li et al. 2024). Here, we briefly summarize the procedures.

### Participants

Sixteen healthy participants (7 male, 9 female) were recruited (age: 23.5 ± 3.4 years, height: 1.71 ± 0.07 m, weight: 64.5 ± 13.2 kg). Participants were recruited only if they did not have any diagnosed orthopedic or neurological disorders and did not use medication that can cause dizziness. Participants were instructed not to engage in intense physical exercise and to refrain from alcohol consumption 24 h prior to the experiment. Two participants were excluded due to technical problems in the signal synchronization. Participants provided written informed consent. The experimental design and procedures were approved by the VU Amsterdam Research Ethics Committee (VCWE-2022-126) and conformed to the Declaration of Helsinki, except for pre-registration.

### Electrical vestibular stimulation

Participants were exposed to a zero-mean stochastic EVS signal with a bandwidth from 0 to 25 Hz, peak amplitude of ± 5.0 mA, and duration of 8 min (Dakin et al. 2010) This signal was applied as an analogue signal through a digital-to-analogue converter (National Instruments Corp., Austin, USA) to an isolated constant-current stimulator (BIOPAC System Inc., Goleta, USA), which was connected to carbon rubber electrodes (9 cm^2^). The electrodes were coated with electrode gel (SonoGel, Bad Camberg, Germany) and were placed over the mastoid processes behind the ears.

### Ground reaction forces and kinematics

Ground reaction forces were measured at 1000 samples/s by force plates embedded within the split-belt treadmill (ForceLink b.v., Culemborg, the Netherlands). Kinematic data were collected using a 3D motion capture system (Northern Digital Inc, Waterloo Ont, Canada) at a sampling rate of 50 samples/s. Cluster markers were attached to the feet, shanks, thighs, pelvis, trunk, head, upper arms and forearms. Corresponding anatomical landmarks were digitized using a six-marker probe based on the model as Kingma et al. (1996).

### Electromyography

A 64-channel REFA amplifier (TMSi, Oldenzaal, The Netherlands, CMRR: > 90 dB, input impedance: > 100 MΩ, gain: 26.55) was used to record the bipolar surface electromyography (EMG) at 2048 samples/s. After shaving, abrading, and cleaning the skin with alcohol, paired disposable self-adhesive Ag/AgCl surface electrodes (Ambu blue sensor, Ballerup, Denmark) were placed 2–3 cm lateral to the spinous processes at eight vertebral levels bilaterally, including the seventh cervical vertebra (C7), the third, fifth, seventh, ninth and twelfth thoracic vertebra (T3, T5, T7, T9, T12) and the second and fourth lumbar vertebra (L2, L4). A reference electrode was placed over the acromion process.

### Protocol

Before the experiment, participants were exposed to vestibular stimulation in a 3 min walk at 2.8 km/h and were instructed to align their steps to the beat of a metronome at 78 steps/min for familiarization. The metronome was used to control the participants’ cadence and to avoid large differences in cadence between the conditions. During the experiment, participants walked with eyes open on the dual-belt treadmill at 2.8 km/h with a cadence of 78 steps/min for 8 min in six conditions. The conditions were defined by the presence of EVS (EVS and no-EVS), by head orientation: head forward (HF) or head leftward (HL) and by step width (preferred step width: PSW and narrow step width: NSW). In the head forward condition, participants were asked to look straight ahead to a target fixed on the wall. In the head facing leftward condition, participants were asked to turn their head to the maximal comfortable position to the left. The order of the conditions was randomized for each participant. Participants received repeated verbal instruction to maintain their cadence and head orientation during the trials. Data from narrow step width conditions were not used in this study.

### Signal analysis

The global coordinate system was defined as positive x laterally to the right, positive y-axis forward, positive z-axis vertically upward. To measure if, and how well participants performed the task of controlling head orientation, cadence and step width, the time series of the head orientation was first calculated using Euler decomposition (z-y-x sequence) in the global coordinate system, then averaged over gait cycles at each normalized time point for each participant. To remove any offset in the local joint coordinate systems, orientations in the head leftward (HL) condition were corrected by subtracting the averaged orientation in walking with head forward without EVS for each participant. In the rest of this paper, we define the head orientation as 0° when head facing forward, positive angles as facing leftward, and negative angles as facing rightward.

The electromyography, force plate and vestibular stimulation signals were synchronized based on trigger signals. Gait events (i.e., heel strikes and toe-offs) were calculated based on the center of pressure (Roerdink et al. 2008). Cadence was calculated from the time between subsequent heel strikes of the same leg.

Step width was calculated as the distance between the second toe tip markers during heel strikes in the mediolateral direction. EMG signals (sampled at 2048 Hz) were resampled to 1000 Hz to align with the sampling rate of the force plate and EVS signals. To remove EVS artifacts, a sixth-order Butterworth high pass filter with a cutoff frequency of 100 Hz was applied on bipolar EMG signals, followed by rectification of the filtered EMG signals (Blouin et al. 2011; Forbes et al. 2014; Li et al. 2024). For each vertebral level and participant, rectified EMG was normalized to the maximum amplitude of the EMG signal of the averaged gait cycle during walking with preferred step width, while facing forward and without EVS. Then, based on right heel strikes, the EVS and normalized EMG signals were sliced into gait cycles. To avoid distortion in the coherence and gain calculation, each gait cycle was padded with data from the previous (50%) and subsequent (50%) cycle. The coherence and frequency response function (FRF) between EVS and EMG were calculated based on the continuous Morlet wavelet decomposition as described in Li et al (2024). The gain (1/milliampere, mA^−1^) and phase estimates (radian, rad) between EVS and EMG were defined as the modulus and the angle of frequency response function, respectively. Phase estimates were transformed into time lags (millisecond, ms) by dividing by their corresponding frequencies. The gain and delay were defined as the median of the gains and time lags across the frequencies and gait phases at peak coherence for each vertebral level and each participant.

### Statistical analysis

To test whether the instructions were followed, repeated-measures ANOVAs with two factors: head orientation (HO: head forward (HF) and head left (HL)) × the presence of EVS (EVS and No EVS) were performed on head orientation (yaw) and cadence. To determine the differences in average muscle activity between conditions, one-dimensional statistical parametric mapping-based repeated-measures ANOVAs were performed with two factors: head orientation × presence of EVS (Pataky et al., 2016). To identify any substantial effects on muscle activity, significance was set as *p* value < 0.01.

Since coherence is naturally bounded between 0 and 1, the modified Fisher-Z transform was applied on coherence values before statistical analysis. For each participant, coherence exceeding 0.015, (i.e., *p* < 0.01), was defined as significant (Zhan et al. 2006). To identify whether the coupling between EVS and EMG significantly differed between head orientations, cluster-based permutation tests, using t-statistics and 2000 permutations, were applied to the coherence (Maris et al. 2007). The effects of head orientation, vertebral level, and side (L: left and R: right side of the trunk), and their interactions on delays and gains were tested with repeated-measures ANOVAs. Sphericity for repeated-measures ANOVAs was verified by Mauchly’s test (*p* > 0.05). Greenhouse–Geisser corrections were applied when the assumption of sphericity was rejected. A Bonferroni correction was applied for the post hoc tests. A *p* value less than 0.05 was defined as significant. Statistical analyses were performed in MATLAB (2019a, The MathWorks, Natick, US).

## Results

**Fig. 1:**
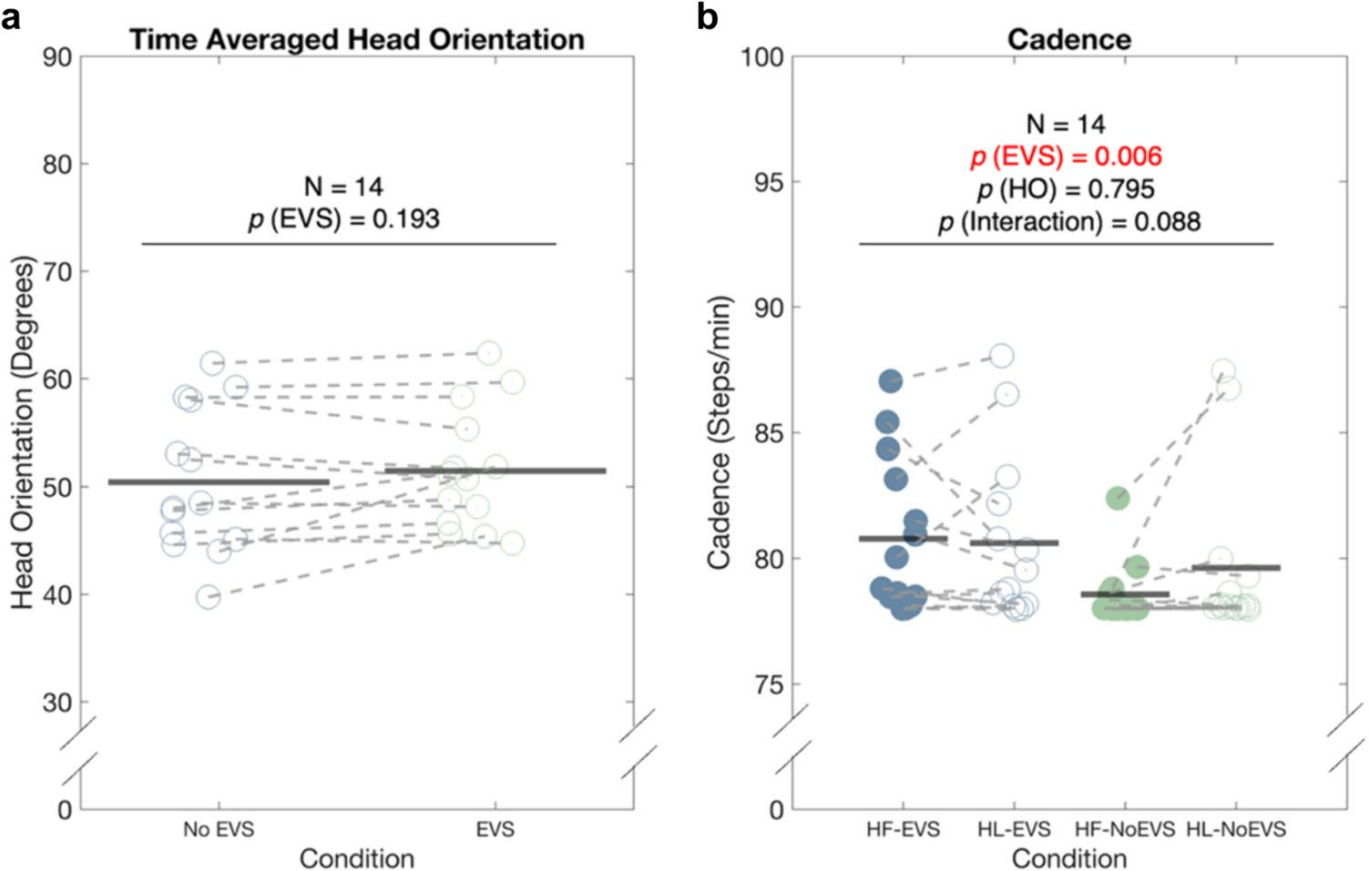
**a** Time-averaged head orientation (in degrees) during walking with EVS (empty green dots) and without EVS (empty blue dots). **b** Time-averaged cadence (in steps per minute) during walking in four conditions: head forward with EVS (HF-EVS, filled blue dots), head leftward with EVS (HL-EVS, empty blue dots), head forward without EVS (HF-No EVS, filed green dots) and head leftward without EVS (HL-No EVS, empty green dots). Each dot represents an individual data point. Black horizontal lines represent the averages across participants

Averaged over time, participants maintained a head orientation of 50.95 (± 6.09) degrees during walking facing leftward (HL) (Fig. 1a). No significant effect of EVS on averaged head orientation was found (*p* = 0.193).

When walking without EVS and facing forward, participants maintained their cadence as instructed (78 steps/min vs 78.57 ± 1.16 steps/min). However, cadence significantly increased with the presence of EVS to 80.78 ± 2.95 steps/min (F (0.763, 9.922) = 10.622, *p* = 0.006). No significant effects of head orientation or its interaction with EVS were found on cadence (Fig. 1b).

Step width was significantly increased by the presence of EVS (F (0.520, 6.760) = 9.293, *p* = 0.009) from 0.16 (± 0.03) m to 0.17 (± 0.04) m when facing forward, and from 0.17 (± 0.05) m to 0.18 (± 0.06) m when facing leftward. No significant effects of head orientation or its interaction with EVS were found on step width.

**Fig. 2:**
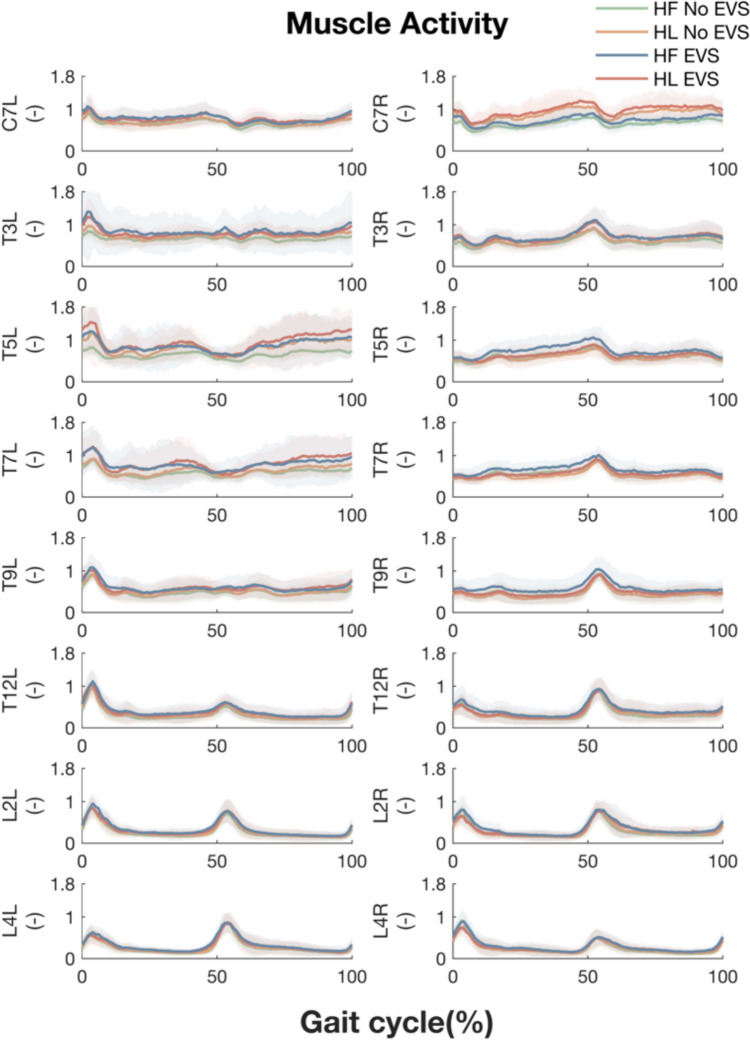
Averaged EMG linear envelopes of paraspinal muscles at all vertebral levels during walking with head forward without EVS (green), with head leftward without EVS (orange), with head forward with EVS (blue) and with head leftward with EVS (red). The left and right columns depict the left and right sides relative to the midline of the trunk. The X-axis is the time (normalized to gait cycle, starting on the right heel strike). The Y-axis indicates the amplitude of the normalized EMG. The lines and shadowed areas represent the means and standard deviations across participants, respectively

Similar phasic activity of the paraspinal muscles was found in walking when facing leftward and forward (Fig. 2). A significant effect of head orientation was only found for muscle activity at the right C7 level around heel strikes (Table 1), likely for maintaining the leftward head orientation during walking. Muscle activity at lower vertebral levels significantly increased with the presence of EVS. No significant effects of the interaction between head orientation and the presence of EVS on muscle activity were found (Table 1). Given these findings, it is reasonable to compare EVS-EMG coherence and gain between the two head orientation conditions below.

**Table 1:** Summary of the effect of the presence of EVS and head orientation on muscle activity at eight vertebral levels on the left (top) and right side (bottom) relative to the trunk

Vertebral level	Presence of EVS		Head orientation		Interaction	
	Phase (% gait cycles)	*p* value	Phase (% gait cycles)	*p* value	Phase (% gait cycles)	*p* value
C7L	/	n.s	/	n.s	/	n.s
T3L	/	n.s	/	n.s	/	n.s
T5L	/	n.s	/	n.s	/	n.s
T7L	/	n.s	/	n.s	/	n.s
T9L	/	n.s	/	n.s	/	n.s
T12L	/	n.s	/	n.s	/	n.s
L2L	20%	0.008	/	n.s	/	n.s
	28%	0.009	/	n.s	/	n.s
	39 ~ 47%	< 0.001	/	n.s	/	n.s
	60% ~ 71%	0.008	/	n.s	/	n.s
L4L	9%	0.009	/	n.s	/	n.s
	28%	0.009	/	n.s	/	n.s
	30 ~ 34%	0.008	/	n.s	/	n.s
	38 ~ 48%	< 0.001	/	n.s	/	n.s
	64%	0.009	/	n.s	/	n.s

Vertebral level	Presence of EVS		Head orientation		Interaction	
	Phase (% gait cycles)	*p* value	Phase (% gait cycles)	*p* value	Phase (% gait cycles)	*p* value
C7R	/	n.s	3%	0.009	/	n.s
	/	n.s	5 ~ 8%	0.004	/	n.s
	/	n.s	9%	0.009	/	n.s
	/	n.s	41 ~ 67%	< 0.001	/	n.s
	/	n.s	87 ~ 96%	< 0.001	/	n.s
T3R	/	n.s	/	n.s	/	n.s
T5R	/	n.s	/	n.s	/	n.s
T7R	/	n.s	/	n.s	/	n.s
T9R	/	n.s	/	n.s	/	n.s
T12R	59 ~ 60%	0.010	/	n.s	/	n.s
	94%	0.010	/	n.s	/	n.s
L2R	35%	0.007	/	n.s	/	n.s
	45%	0.006	/	n.s	/	n.s
	69 ~ 73%	0.003	/	n.s	/	n.s
	84%	0.008	/	n.s	/	n.s
	89%	0.010	/	n.s	/	n.s
	91 ~ 97%	< 0.001	/	n.s	/	n.s
L4R	8 ~ 11%	0.007	/	n.s	/	n.s
	46 ~ 49%	0.004	/	n.s	/	n.s
	91%	0.010	/	n.s	/	n.s

Results from the one-dimensional statistical parametric mapping-based repeated-measures two-ways ANOVAs. The *p* values indicate the probability that a suprathreshold cluster of the same spatiotemporal extent could have resulted from a random field process of the same smoothness as the observed residuals

### Phasic EVS-EMG coupling in walking with head leftward

**Fig. 3:**
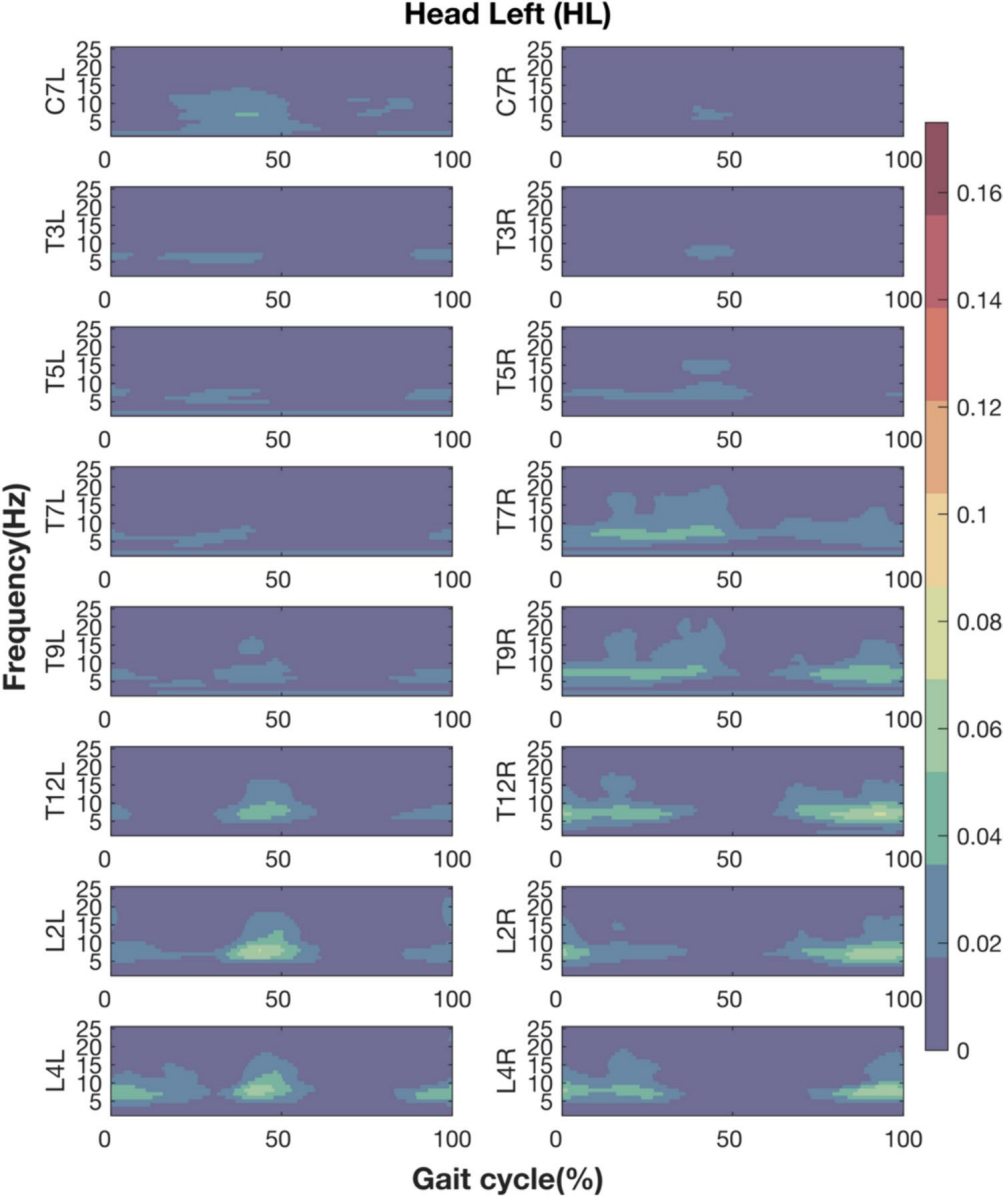
Coherence between EVS and EMG during walking with head leftward. Figures from top to bottom rows present the results at eight vertebral levels. The left and right columns depict the left and right sides relative to the midline of the trunk. The X-axis is the time (normalized to gait cycle, starting at the right heel strike). The Y-axis is the frequency ranging from 1 to 25 Hz. The magnitudes of coherence are indicated by the color bar

The spatiotemporal pattern of EVS-EMG coherence was similar between walking with head forward (Li et al. 2024) and leftward. Significant EVS-EMG coherence was observed at all recorded sites and peaked around ipsilateral heel strike at lower vertebral levels and at contralateral heel strike at higher vertebral levels (Fig. 3).

### Decrease in EVS-EMG coupling induced by head orientation

**Fig. 4:**
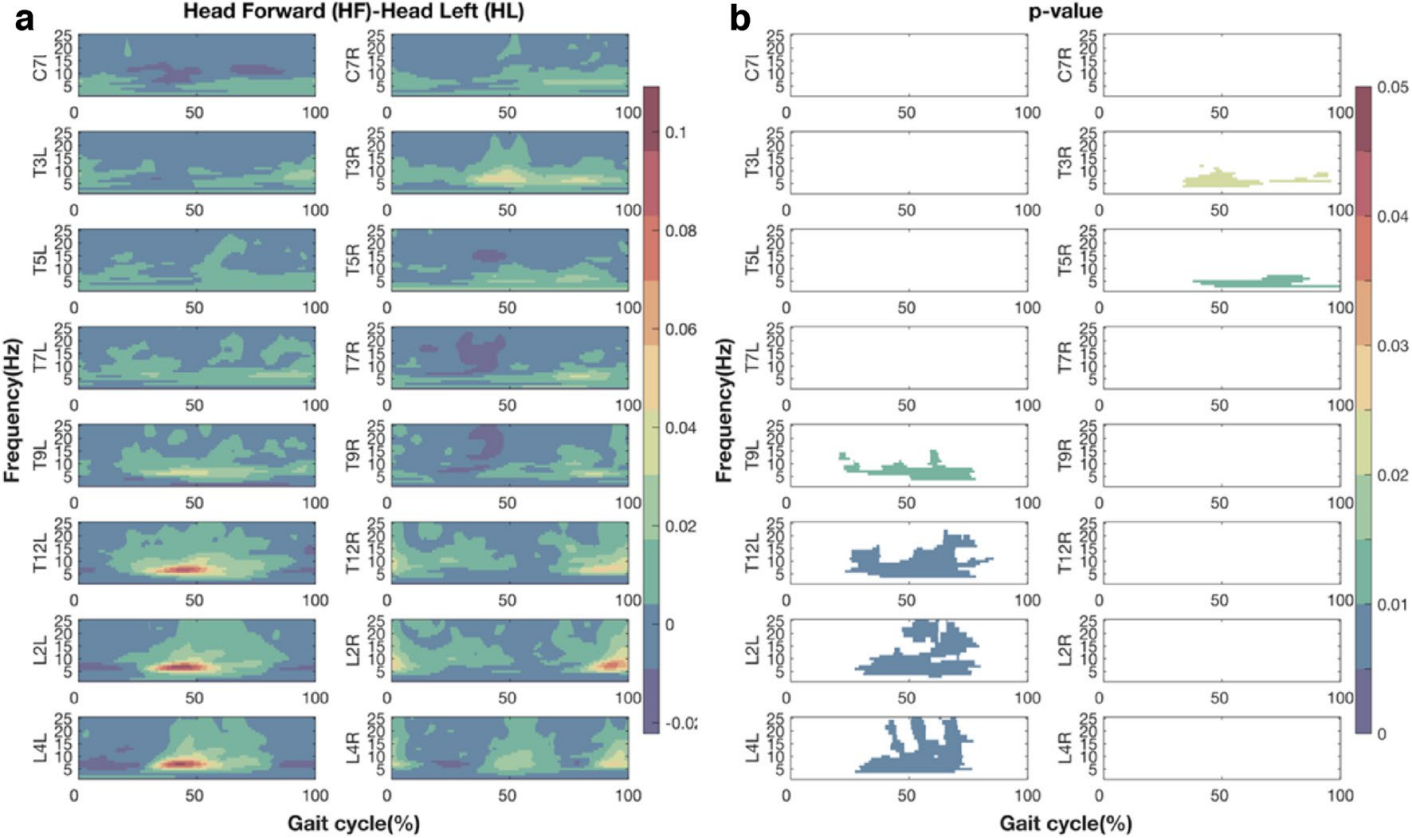
The differences in coherence (**a**) and associated *p* values (**b**) between two head orientations (head forward - head leftward) during walking with EVS. Figures from top to bottom rows present the results at eight vertebral levels. The left or right columns represent the left or right sides relative to the mid-sagittal line of trunk. The X-axis is the time (normalized to gait cycle, starting on the right heel strike). The Y-axis is the frequency ranging from 1 to 25 Hz. The magnitudes are indicated by the color bar. For clarity, only *p* values lower than 0.05 are presented

When walking facing leftward, EVS-EMG coupling significantly decreased around left heel strike at vertebral levels from T9 to L4 on the left side of the trunk and at vertebral levels from T3 to T5 level on the right side of the trunk (Fig. 4).

### Asymmetric EVS-EMG coupling between the left and right sides of the trunk

**Fig. 5:**
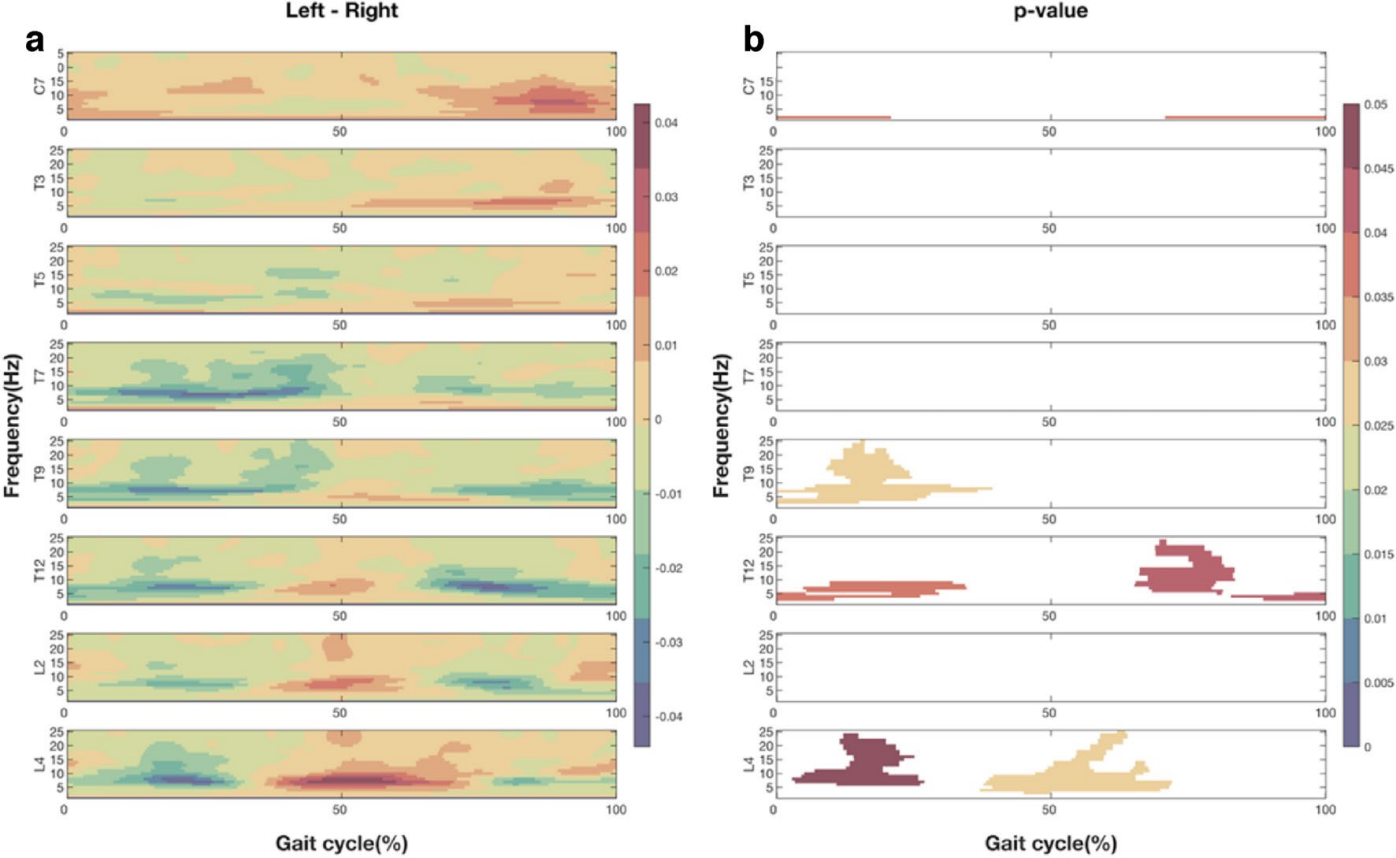
The differences in coherence (**a**) and associated *p* values (**b**) between EVS and EMG during walking with head leftward between the left and right sides of trunk (left–right). Figures from top to bottom rows present the results at eight vertebral levels. The X-axis is the time (normalized to gait cycle, starting at ipsilateral heel strike). The Y-axis is the frequency ranging from 1 to 25 Hz. The magnitudes are indicated by the color bar. For clarity, only *p* values lower than 0.05 are presented

To further explore the asymmetric decrease in EVS-EMG coherence during walking with the head turned to the left, statistical parametric mapping-based paired t-tests were performed on the EVS-EMG coherence between the left and right sides of the trunk, aligned at ipsilateral heel strikes (Fig. 5a). During walking with the head turned left, EVS-EMG coherence was significantly higher on the left at C7 in early ipsilateral stance and late contralateral stance, and at L4 around contralateral heel strike. However, coherence was significantly lower at T9 and T12 during ipsilateral stance, and during contralateral stances at T12 (Fig. 5b). Taken together, these effects confirm the asymmetric effects of head rotation on EVS-EMG coupling.

### Leftward head orientation induced decrease in gain

**Fig. 6:**
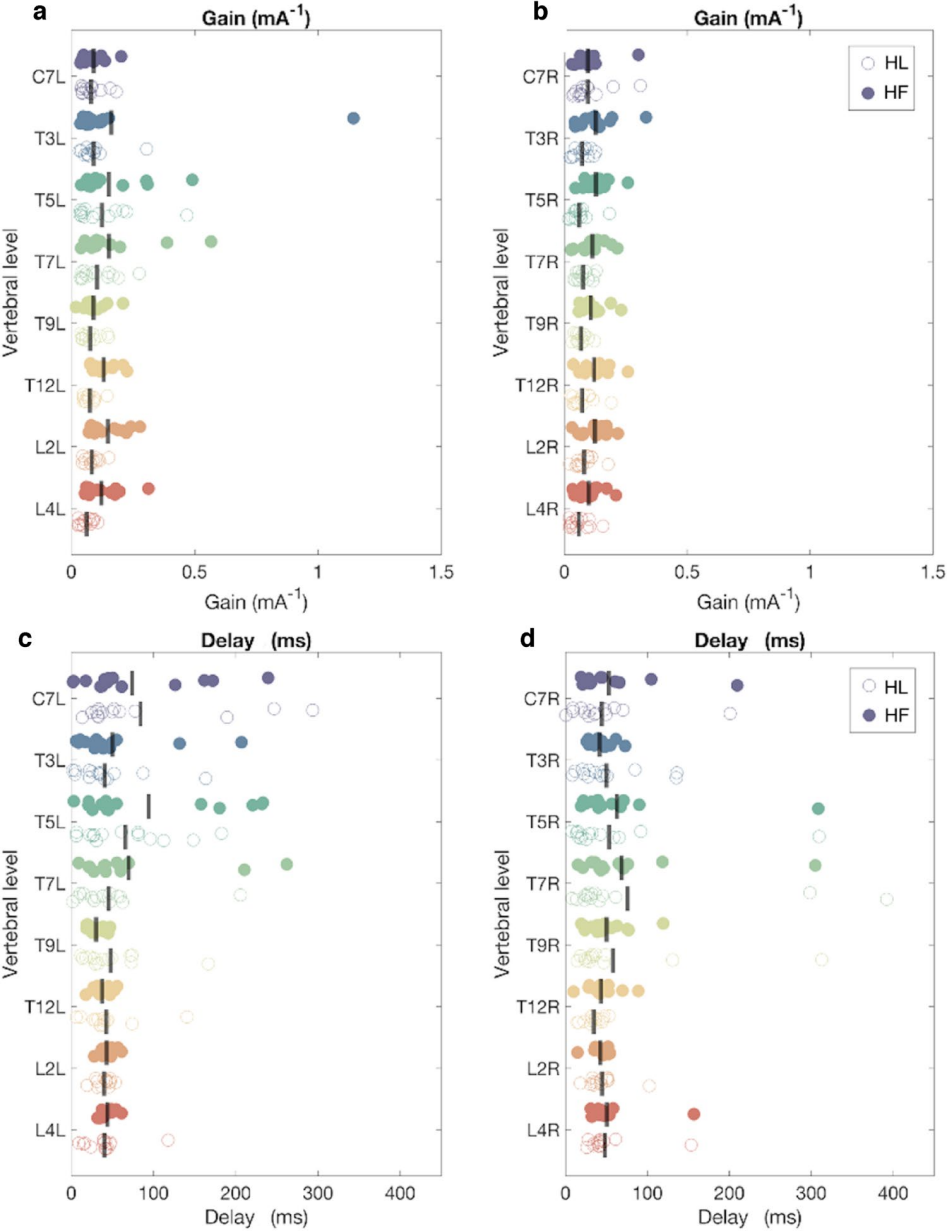
Gain and delay estimates between EVS and EMG. The Y-axis represents vertebral levels (C7, T3, T5, T7, T9, T12, L2 and L4) on the left (**a**, **c**) and right side (**b**, **d**) of the trunk. In panel A and B, the X-axis represents the magnitude of the gain (mA.^−1^). In Panel C and D, the X-axis represents the delay (ms) between EVS and EMG. Each filled dot indicates the individual data during walking with head forward (HF). Each empty dot indicates the individual data during walking with head leftward (HL)

Gain was significantly higher when walking facing forward than facing leftward (F (0.079, 1.027) = 12.883, *p* = 0.003) (Fig. 6A, B; Table 2). No significant effect of either vertebral level, side nor of any of the interactions was found on gain.

**Table 2:** Summary of the three-way ANOVA test (head orientation × vertebral levels × side)

Gain	*N*	*F*	*p*
Vertebral levels	14	F (0.553, 7.189) = 1.056	0.372
Head orientations	14	F (0.079,1.027) = 12.883	0.003
Sides	14	F (0.079,1.027) = 1.483	0.245
Vertebral levels × head orientations	14	F (0.553, 7.189) = 1.840	0.190
Vertebral levels × sides	14	F (0.553, 7.189) = 1.035	0.386
Head orientations × sides	14	F (0.079,1.027) = 0.018	0.895
Vertebral levels × head orientations × sides	14	F (0.553, 7.189) = 1.598	0.213

Phase	*N*	*F*	*p*
Vertebral levels	14	F (1.379, 17.927) = 2.194	0.087
Head orientations	14	F (0.197, 2.561) = 0.161	0.695
Sides	14	F (0.197, 2.561) = 0.091	0.768
Vertebral levels × head orientations	14	F (1.379, 17.927) = 0.423	0.753
Vertebral levels × sides	14	F (1.379, 17.927) = 1.409	0.257
Head orientations × sides	14	F (0.197, 2.561) = 0.345	0.567
Vertebral levels × head orientations × sides	14	F (1.379, 17.927) = 0.717	0.559

### No effect of head orientation on delays

As expected, no significant effects on delays of head orientation, vertebral level, side or any of the interactions were found (Fig. 6C, D; Table 2).

## Discussion

Using stochastic EVS, we compared how vestibular signals influence paraspinal muscle activity, from cervical to lumbar level, while walking with the head facing forward and sideways, respectively. Significant phasic coherence between the EVS and paraspinal muscle activity was observed when walking with the head facing leftward as during walking with head forward, i.e., the coherence peaked at contralateral heel strike at higher vertebral levels, whereas it peaked at ipsilateral heel strike at lower vertebral levels. The gains and coherences between EVS and EMG significantly decreased when walking with the head facing leftward. The decrease in EVS-EMG coherence was asymmetric between the left and right sides of the trunk, i.e., the decrease was significant around left heel strikes at the left side between T9 and L4 and at the right side between T3 and T5. No effect of head orientation, vertebral level, side of the trunk nor their interaction was found on the delays of the EMG responses. These results agree with the idea that less feedback control is required in the sagittal plane to stabilize walking. However, it does not explain the asymmetric nature of the decrease in coherence when walking with the head sideways.

### Decreases in coherence and gain suggest less feedback control in the sagittal plane

When standing with head facing rightward, EVS with the anode on the right mastoid process induces a backward sway as the response to an illusion of forward sway (Lund and Broberg 1983; Britton et al. 1993; Ali et al. 2003; Mian and Day 2014; Forbes et al. 2016). Underlying this backward sway, bilateral activation of posterior lower limbs and erectors spinae muscles was observed (Britton et al. 1993; Ali et al. 2003). Based on this, we expected a more symmetric spatiotemporal pattern of EVS-EMG coherence in paraspinal muscles during walking with the head facing sideways. In contrast, phasic EVS-EMG coherence with a significant decrease in magnitude was found when walking with the head facing to the left. Combined with the decrease in gains, the overall decrease indicates less reliance on vestibular signal for the control of the trunk muscles in the sagittal plane than in the frontal plane during walking. This is likely be related to less reliance on feedback control in the sagittal plane, which is a consequence of the larger length than width of the base of support in walking and the fact that passively determined foot placement can stabilize gait in the sagittal, but not in the frontal plane (Bauby and Kuo 2000; O'Connor and Kuo 2009). When the head is turned, vestibular afferent signals partly encode movements in the sagittal plane, hence responses to EVS decrease compared to when facing forward. As reported in standing by Lund et al. (1983), with a head rotation of − 60°, EVS with the anode on the right, induced body sway in a direction of about − 150°. Thus, the remaining phasic EVS-EMG coupling pattern is possibly due to the projection of the disturbance signal on the frontal plane caused by the head rotation less than 90° (Lund and Broberg 1983; Britton et al. 1993; Ali et al. 2003).

In addition, head orientation can be a risk factor of falling during walking. Though vestibular exafference is continuously reinterpreted for adjusting motor output with head orientation changes, suppression of EVS-induced response was found during voluntary head movement (Osler and Reynolds 2012). With the reduced vestibular information input in the frontal plane induced by sideway head orientation, these indicate the possibility of impaired walking stability control by head orientation.

### Asymmetry in the decrease in EMG-EVS coherence when walking while facing leftward

Asymmetric suppression in EVS-EMG coherence was observed between the left and right sides of the trunk when walking with the head leftward. This is consistent with previous studies in lower limb muscles during standing (Britton et al. 1993; Ali et al. 2003; Dakin et al. 2007; Forbes et al. 2016). This asymmetric response was ascribed to the thorax rotation used to reach the large degree of head rotation without discomfort (Britton et al. 1993). Indeed, an averaged 5° thorax rotation also exists in our study (HL-EVS: 5.19 ± 2.24°, HL-No EVS: 4.89 ± 1.94°, results not shown). This causes a biomechanical asymmetry, which possibly modifies the EVS induced activation of the paraspinal muscles.

**Fig. 7:**
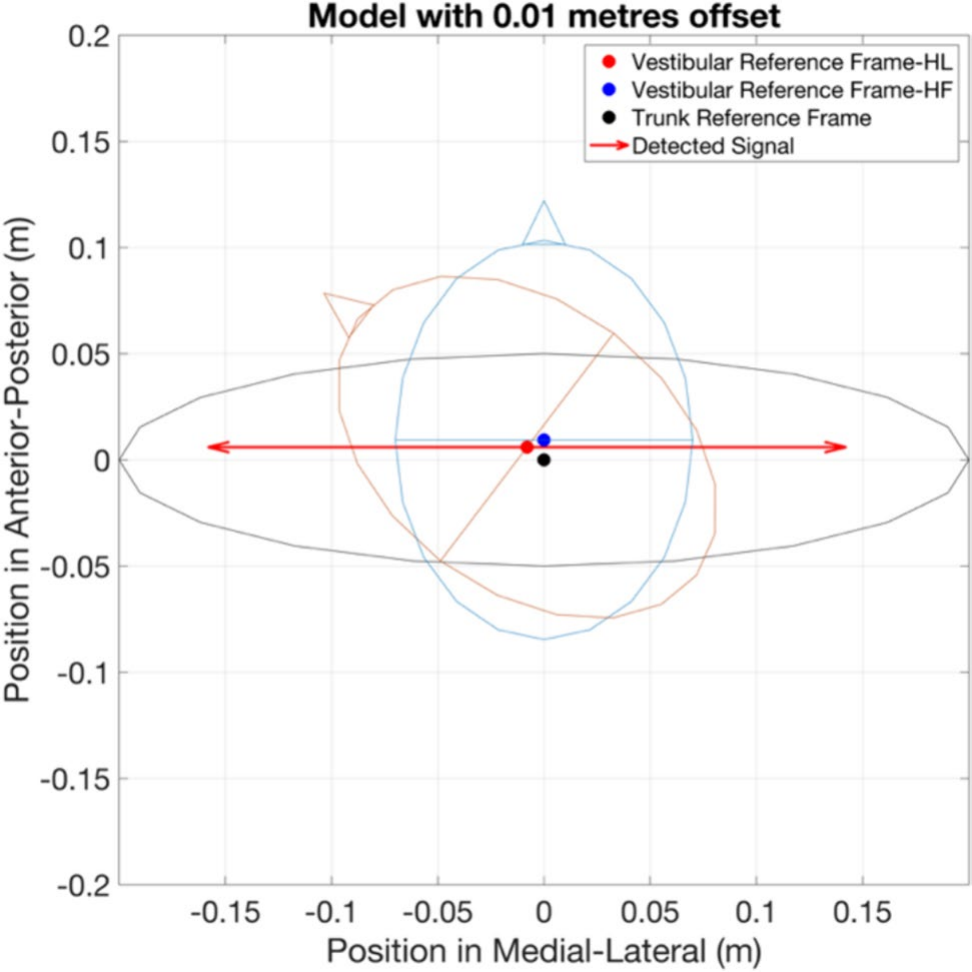
The head-fixed vestibular reference frame related to the orientation of the trunk (black circle) when facing forward (blue circle) and with the head turned 50° (red circle). The X and Y-axes represent the position on the medial–lateral and anterior–posterior axes, in meters, respectively. Filled dots indicate the origin of trunk reference frame (in black), and of the vestibular reference frame when head facing forward (in blue) and facing leftward (in red). Vectors indicate the vestibular signals encoding illusions with the amplitude of 0.15 m to both the left and right, detected in the vestibular reference frame with offset (detected signal, in red). With the offset, the projection of detected signals into the trunk reference frame induces an increased leftward illusion and a decreased rightward illusion. Consequently, compensatory responses to overcome the illusions are asymmetric in magnitude

Another potential explanation is that head turn induced an asymmetric projection of the vestibular signal in the reference frame of the trunk. The midpoint of the interaural axis between the vestibular labyrinths is assumed to form the origin of the vestibular reference frame in the transverse plane (Moore et al. 2005). However, the yaw axis of head rotation is located close to the atlanto-occipital and atlanto-axial joints, which are posterior to the vestibular labyrinths. Previously, in standing and sitting, the axis of yaw rotation was reported to be located about 10 mm posterior to the midpoint between the vestibular labyrinths (Moore et al. 2005). With this distance, an offset of the vestibular axis to the left relative to the trunk reference frame will be induced (Fig. 7). In this scheme, the vestibular signal encoding leftward motion would be reduced more than those encoding movement to the right, causing an asymmetric response to vestibular afference.

## Limitations

A limitation in this study is that the averaged head rotation did not reach 90°, with which EVS would produce a disturbance purely in the sagittal plane (Forbes et al. 2016; Tisserand et al. 2018). However, as reported elsewhere, the maximal range of head yaw rotation is between ± 60° to ± 80° with slight thorax and/or pelvis rotation in standing (Ferrario et al. 2002; Tommasi et al. 2009; Higgins et al. 2023). The test conditions, therefore, reflect a feasible range of rotation. In addition, only head leftward condition was included, which provides a limited view of the effect of head orientation on vestibular signal-based modulation of paraspinal muscle activity. Future investigations should include more orientations in both directions as was done for standing in Forbes et al (2016).

In addition, changes in the gait parameters, i.e., cadence and step width, can indicate the use of different more generic stabilization strategies, i.e., walking with higher cadence or wider steps. Thus, we controlled these variables by repeated verbal instruction, metronome and projected lines (only in head forward conditions). Nevertheless, we found that EVS increased the cadence and step width. This provides a more robust gait pattern and thus decreased demands on active stability control. Dakin et al (2013) observed decreased EVS-EMG coherence in some lower limb muscles, when walking at an increased cadence. However, in Dakin’s study, the comparison was between cadences of 52 and 78 steps/min. In our study, the presence of EVS increased the cadence with a relatively small magnitude from 78.57 to 80.78 steps/min in head forward condition and 79.62–80.61 steps/min in head leftward condition. Moreover, the cadence was very similar between head orientation conditions. Thus, we think that our main comparison between head orientations will not be affected.

Another limitation is that measurements were done on a treadmill instead of during overground walking. The literature has shown that spatiotemporal, kinematic, kinetic, electromyographic and energy consumption outcome measures are comparable between treadmill walking and overground walking (van Ingen, 1980; Martin and Li 2017; Semaan et al. 2022; Jung and Koo 2022). However, variability of gait kinematics appears to be lower in treadmill walking, presumably due to a constraint on fluctuations in velocity (Dingwell et al. 2000). More importantly, a prominent difference between overground and treadmill walking is the absence of visual flow in treadmill walking. Sylcott et al. (2021) reported an increased activation in the prefrontal and vestibular cortex during walking on the treadmill compared to walking overground. This suggests that during treadmill walking, sensory reweighting takes place and vestibular information is likely upweighted.

## Conclusion

When participants walked with their head facing leftward, significant phasic EVS-EMG coherence in paraspinal muscles was observed at ipsilateral and/or contralateral heel strikes, similar to when facing forward. Significant decreases in stimulation-evoked responses (i.e., EVS-EMG coherence and gain) in paraspinal muscles were observed, when walking facing left compared to facing forward. This can possibly be explained by less need of the feedback control to stabilize walking in the sagittal plane compared to the frontal plane. The decrease in coherence was only significant around left heel strikes at the left side between T9 and L4 and at the right side between T3 and T5. The directionally specific response is likely be to a consequence of the offset between the origin of vestibular reference frame to head yaw rotation pivot. In sum, our findings confirmed the contribution of vestibular signal in trunk stabilization, while highlighting that this contribution is impacted by head orientation.

## Data Availability

The original data are available from the corresponding author upon reasonable request.

## Abbreviations

ANOVA: Analysis of variance
C: Cervical vertebrae
EMG: Electromyography
EVS: Electrical vestibular stimulation
HF: Head forward
HL: Head leftward
HO: Head orientation
L: Lumbar vertebrae
N.S.: Not significant
T: Thoracic vertebrae
